# Efficacy of Two Estrus Synchronization Protocols in Crossbred Gyr Dairy Cows and Their Relationship with Heat Stress in the Peruvian Tropics

**DOI:** 10.3390/vetsci12090804

**Published:** 2025-08-25

**Authors:** Ronald W. Vásquez-Tarrillo, José E. Hernández-Guevara, Benjamín A. Depaz-Hizo, Gustavo Ampuero-Trigoso, Annie Y. Poclín-Rojas, Marisol Rojas-Reategui, Gleni T. Segura Portocarrero, Jorge D. Juarez-Moreno, Hurley A. Quispe-Ccasa

**Affiliations:** 1 Agricultural Experimental Station El Porvenir, Instituto Nacional de Innovación Agraria, Juan Guerra 22400, Peru; rvasqueztarrillo0609@gmail.com (R.W.V.-T.); bdepaz@inia.gob.pe (B.A.D.-H.); anniepoclinrojitas@gmail.com (A.Y.P.-R.); mariolita789@gmail.com (M.R.-R.); 2 Facultad de Zootecnia, Universidad Nacional Agraria de la Selva, Tingo María 10131, Peru; jose.hernandez@unas.edu.pe (J.E.H.-G.); jorge.juarez@unas.edu.pe (J.D.J.-M.); 3 Dirección de Desarrollo Tecnológico Agrario, Instituto Nacional de Innovación Agraria, Lima 2791, Peru; gustavoampuerotrigoso@gmail.com; 4 Facultad de Ingeniería Zootecnista, Agronegocios y Biotecnología, Universidad Nacional Toribio Rodríguez de Mendoza de Amazonas, Chachapoyas 01001, Peru

**Keywords:** tropical livestock, estrus synchronization, prolonged proestrus, Gyr, THI

## Abstract

The performance of estrus synchronization protocols may be influenced by stressful environmental conditions, even in tropically adapted cattle. This study evaluated the efficacy of two synchronization protocols for fixed-time artificial insemination (FTAI) in crossbred Gyr dairy cows and their relationship with heat stress under Peruvian tropical conditions. Multiparous lactating cows under semi-intensive systems were synchronized and divided into two groups: the conventional protocol (PC9) and the prolonged proestrus protocol (J-Synch6). Estrus manifestation, pregnancy rate, and their association with the temperature–humidity index (THI) were analyzed across seven phases of the FTAI program (73 days). In PC9 cows, no significant effect of the number of hours in each heat stress category according to THI was found. Conversely, in J-Synch6 cows, more hours in Category 0 (THI < 72) during Phases 1 to 4 (follicular emergence to preovulatory follicle) had a positive effect on the estrus manifestation, whereas in Phase 3 (follicular growth), higher exposure to Category 3 (THI ≥ 83) had a negative effect on estrus manifestation. Both protocols demonstrated similar estrus and pregnancy rates, although heat stress affected estrus rates in J-Synch6, but not pregnancy. The choice of protocol should account for heat stress risks during specific periods of the year to maximize success.

## Simple Summary

Reproductive efficiency may be influenced by heat stress, even in tropical cattle. This study evaluated the efficacy of two estrus synchronization protocols for fixed-time artificial insemination in 159 Gyr dairy cows divided into two synchronization groups: a conventional protocol and a J-Synch6 protocol. No significant differences were found in estrus or pregnancy rates between groups. In the J-Synch6 protocol, more hours in a no-stress state during antral follicle, onset of the previous estrus cycle, follicular growth, and pre-ovulatory follicle phases improved the probability of estrus manifestations, and a higher exposure to an emergency state of heat stress in the follicular growth phase negatively affected the estrus signs. The choice of protocol should account for heat stress risks during specific periods of the year to maximize success in tropical conditions.

## 1. Introduction

Reproductive efficiency in tropical cattle is essential to ensure the sustainability of livestock breeding [1]. Gyr cattle and their crossbreeds are known for their high productive efficiency in warm climates and disease resistance, making them a valuable option for milk production [2]. However, environmental conditions, particularly high temperatures, can still negatively affect estrus expression and pregnancy rates [3], even in adapted breeds. These conditions limit cattle’s reproductive health and potential due to metabolic alterations and dysfunctions in neuroendocrine reproductive regulation [4]. Heat stress can lead to anovulation, reduced oocyte quality, decreased uterine receptivity, and lower fertility [5,6].

Zebu breeds face significant challenges in improving reproductive performance, including the lack of evident estrus signs, making timely heat detection difficult [7]. Reproductive biotechnologies have optimized efficiency in cattle by improving genetic quality, productivity, and herd profitability. Fixed-time artificial insemination (FTAI) is an effective tool for improving pregnancy rates in cattle by eliminating the need for heat detection and allowing precise service planning [8]. This is particularly relevant in production systems with infrastructure deficiencies and heat detection capacity [9].

Ovulation synchronization through the administration of exogenous hormones is essential for FTAI, and its success relies on selecting the appropriate hormonal protocol [10]. The conventional protocol involves the use of progesterone, estradiol, and prostaglandins to synchronize the estrous cycle for optimal timing of insemination, maximizing pregnancy rates [11]. However, the use of estradiol salts at the time of progesterone device removal shortens the proestrus phase. Consequently, the dominant follicle does not reach its full growth potential before ovulation, resulting in a smaller pre-ovulatory follicle, lower oocyte quality, and reduced pregnancy rates [12,13,14]. The J-Synch protocol extends the proestrus phase, allowing the dominant follicle more time to complete its development, reaching diameters of 12 to 14 mm before ovulation [15]. Prolonged proestrus may improve oocyte quality and uterine receptivity, increasing fertilization rates [16].

Under tropical conditions, the effectiveness of synchronization protocols may be influenced by heat stress. Our hypothesis was that heat stress could differentially affect estrus expression and pregnancy rates depending on the synchronization protocol used. Therefore, the objective of this study was to evaluate the efficacy of estrus synchronization protocols in crossbred Gyr dairy cows and to relate it to heat stress estimated by the temperature–humidity index (THI) in a FTAI program.

## 2. Materials and Methods

**Figure 1:**
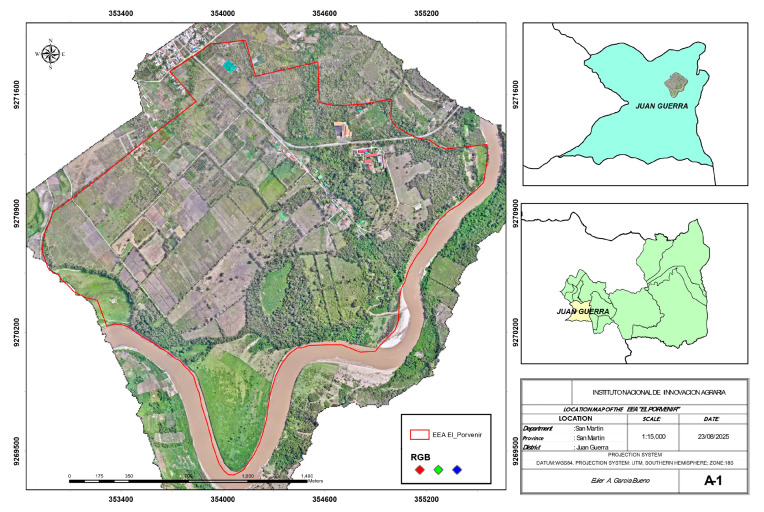
Geographic location map of the study at the Estación Experimental Agraria El Porvenir, San Martín department, Peru.

The study was conducted at the Estación Experimental Agraria “El Porvenir” of the Instituto Nacional de Innovación Agraria, located in the department of San Martín, Peru. The region is situated between coordinates 6°35′6″ S 76°19′10″ W to the north, 6°36′30″ S 76°19′21″ W to the south, 6°35′30″ S 76°18′24″ W to the east, and 6°35′44″ S 76°19′48″ W to the west, at 225 m above sea level (Figure 1). The climate is classified as Humid Tropics according to Köppen–Geiger, with an average temperature of 25 °C, relative humidity of 72.9%, and annual precipitation ranging from 790 to 1200 mm/m^2^. This study was carried out under the approval letter of the university ethics committee No. 002-2025-COMÍTÉ DE ÉTICA-UNAS.

### 2.1. Animals

A sample of 159 crossbred Gyr cows from 4 to 8 years old, with body weight between 350 and 380 kg, body condition score between 2.5 and 3.5 (scale from 1 to 5), and milking with calf at foot, were selected. The cows were managed under a semi-intensive system with grazing on *Brachiaria brizantha*, *Brachiaria decumbens*, and natural pastures (*Axonopus scoparius* and *Paspalum conjugatum*) during two daily periods (6:00 to 11:00 a.m. and 2:30 to 6:00 p.m.). They had ad libitum drinking water and were supplemented with 2 kg/day of a balanced feed (rice bran, corn, soybean meal, and mineral salts).

### 2.2. Experimental Design

The study followed a randomized block design with two FTAI protocols, a conventional protocol (PC9) and a J-Synch6 protocol (J-Synch6), applied across four blocks. The blocks corresponded to four periods in order to optimize the homogeneous application of the protocols in manageable groups, and to control the possible seasonal effect. Lactating crossbred Gyr cows were selected, considering their being at least 60 days postpartum, a body condition score between 2.5 and 3.5, normal ovarian activity presence of a corpus luteum or dominant follicle), and a health history free of recent metabolic (ketosis, hypocalcemia) or reproductive disorders (postpartum anestrus, ovarian cysts) as inclusion criteria. The clinical and gynecological evaluation was performed by an experienced veterinarian using rectal palpation and transrectal ultrasound (Draminski IScan ultrasound scanner), complemented by a review of recent health and reproductive records. Cows were randomly and evenly assigned to the two experimental groups using a random number table generated in an Excel spreadsheet, based on each animal’s identification number (ear tag). However, the number of cows per period depended on the homogeneous gynecological status of the animals at the time of selection. In some formed groups, one or two cows lost their intravaginal device during the protocol and were therefore excluded from subsequent analyses to avoid bias in the results. In Period 1 (September–December), 21 cows were assigned to PC9 and 21 to J-Synch6; in Period 2 (January–March), 13 cows to PC9 and 15 to J-Synch6; in Period 3 (April–July), 23 cows to PC9 and 22 to J-Synch6; and in Period 4 (July–October), 22 cows to PC9 and 22 to J-Synch6, totaling 79 cows to PC9 and 80 cows to J-Synch6.

### 2.3. Estrus Synchronization Protocols

**Figure 2:**
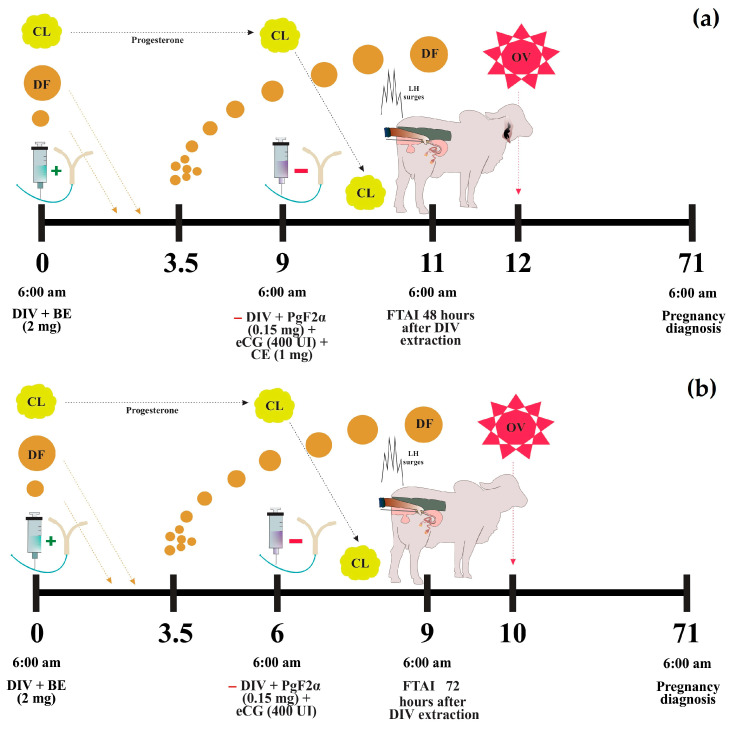
Protocols of estrus synchronization. Conventional protocol or PC9 (**a**) and J-Synch protocol or J-Synch6 (**b**). DIV: intravaginal progesterone device, BE: estradiol benzoate, CE: estradiol cypionate, CL: corpus luteum, DF: dominant follicle, OV: ovulation, FTAI: fixed-time artificial insemination. The (+) sign indicates addition and the (−) sign indicates withdrawal.

Prior to synchronization, all cows were dewormed (Dectomax, Zoetis, Buenos Aires, Argentina) at a dose of 1 mL/50 kg, supplemented with vitamins ADE (Vigantol, Bayer, Leverkusen, Germany) at 5 mL/cow, and minerals (Vitasel, Servinsumos, Bogota, Colombia) at 15 mL/cow. An initial ultrasonographic evaluation was performed on Day 0 to verify reproductive health and the ovarian estrous status. In PC9 protocol: On Day 0, a 1.2 g intravaginal progesterone device (DIV) (Disposint^®^ 1200, León Pharma, Santa Fe, Argentina) and 2 mg of estradiol benzoate (2 mg Estraben^®^, León Pharma, Santa Fe, Argentina) were administered intramuscularly. On Day 9, the DIV was removed, followed by the administration of 0.150 mg of sodium cloprostenol (2 mL D-tenol^®^, León Pharma, Santa Fe, Argentina), 400 IU of eCG (2 mL Novormon^®^ 5000, Zoetis, Buenos Aires, Argentina), and 1 mg of estradiol cypionate (1 mL Estracip^®^, León Pharma, Santa Fe, Argentina) intramuscularly, and an estrus detection patch was also applied. Artificial insemination (AI) was performed 48 h after DIV removal on Day 11 (Figure 2a). In J-Synch6 protocol: On Day 0, a 1.2 g DIV and 2 mg of estradiol benzoate (2 mg Estroben^®^) were administered intramuscularly. On Day 6, the DIV was removed, and 0.150 mg of sodium cloprostenol (2 mL D-tenol^®^) and 400 IU of eCG (2 mL Novormon^®^ 5000) were administered intramuscularly, along with the application of an estrus detection patch. On Day 9, 72 h after DIV removal, AI was performed. Additionally, 0.105 g of Buserelin acetate (2.5 mL Gestar^®^, OVER S.R.L., Santa Fe, Argentina) was administered to cows that did not exhibit signs of estrus (Figure 2b).

### 2.4. Estrus Detection Signs and Artificial Insemination

Estrus detection was performed using ESTROTECT heat detection patches (ESTROTECT ^TM^, Spring Valley, WI, USA), placed transversely between the hips and the base of the tail. The silver and black surface of the patches was removed when cows were mounted by others, revealing a bright red background. Cows were considered to be in estrus if the patch showed ≥75% discoloration. Additionally, cervical mucus discharge and uterine tone (UT) were recorded before artificial insemination on a scale of 1 (most flaccid) to 3 (most turgid), by the same professional with proven experience in estrus synchronization protocols in cattle and who did not know the treatment group to which each cow was subjected to minimize bias in the assessment. Fixed-time artificial insemination (FTAI) was performed using thawed semen from Gyr bulls. After thawing at 37 °C, individual sperm motility (>40%) was verified to ensure semen quality. All inseminations were conducted by specialized technical personnel. Pregnancy diagnosis was performed 60 days after AI by ultrasonography (iScan, Draminski, Gietrzwałd, Poland). The evaluation focused on the presence of embryonic structures and confirmation of fetal heartbeat.

### 2.5. Temperature–Humidity Index (THI)

The temperature–humidity index (THI) was calculated using ambient temperature (Tα) in °C and relative humidity (RH), obtained from the El Porvenir Meteorological Station [17] between September 2021 and August 2022, by the following equation [18]:

$$THI=\left(1.8\;*\;T\alpha +32\right)-[\;\left(0.55-0.55\;*\;\frac{RH}{100}\right)\;*\;\left(1.8\;*\;T\alpha -26\right)\;]$$

THI values were calculated and classified into the following categories: Category 0 or no stress (THI < 72), Category 1 or alert status (THI between 72 and 78), Category 2 or danger status (THI between 78 and 82), and Category 3 or emergency status (THI ≥ 83) [19]. Subsequently, the number of hours spent in each THI maximum category was counted according to seven critical phases of the FTAI program [20]. Phase 1: Day 42 to 1 before AI (antral follicle emergency); Phase 2: Day 21 to 1 before AI (beginning of the previous estrous cycle); Phase 3: Day 2 to 1 before AI (follicular growth); Phase 4: On the day of AI (pre-ovulatory follicle); Phase 5: Day 1 to 3 after AI (ovulation and fertilization); Phase 6: Day 1 to 21 after AI (maternal recognition and implantation); and Phase 7: Day 1 to 31 after AI (early fetal development).

### 2.6. Statistical Analysis

The data were organized in tables to present the categorical variables as absolute frequencies and percentages, while the continuous variable (follicular diameter) was expressed as the mean ± standard deviation. The Chi-square test (*p* < 0.05) was used to analyze the effect of the protocols on estrus signs and pregnancy rate. Follicular diameter was tested for normality (Shapiro–Wilk) and homogeneity (Levene), and subsequently analyzed by ANOVA with the *t*-test (*p* < 0.01). The effect of the number of hours spent in each THI category during each phase of the FTAI program and the binomial response variables were analyzed using a logistic regression in SPSS v.15.0 software.

## 3. Results

The effect of two estrus synchronization protocols on uterine tone (UT), presence of cervical mucus discharge and estrus detection via patches, was evaluated in 159 cows. As shown in Table 1, there was no significant association between synchronization protocol and estrus signs (*p* > 0.05). However, pre-insemination follicular diameter was larger in J-Synch6 (14.27 mm) compared to PC9 (12.54 mm) (*p* < 0.05).

Estrus manifestation and pregnancy rates were further analyzed according to the period, presence of cervical mucus discharge, and initial corpus luteum presence (Table 2). No significant association was found between the estrus rates or pregnancy rates according to periods or according to the presence or absence of cervical mucus (*p* > 0.05). Cows exhibiting cervical mucus discharge were not associated with estrus (53.3–68.1%) and pregnancy rates (34.4–49.3%). The initial corpus luteum presence or absence showed similar estrus manifestation (*p* > 0.05), but a significantly higher pregnancy rate was associated with the corpus luteum presence (*p* < 0.01).

Correlation analysis was performed among estrus signs, pregnancy rate, and initial corpus luteum presence according to the synchronization protocol (Table 3). In both protocols, uterine tone, estrus manifestation, and positive pregnancy were directly correlated (*p* < 0.01), with slightly higher correlation values in J-Synch6. The presence of a corpus luteum was also positively correlated with pregnancy success (*p* < 0.05), showing similar correlation coefficients in both protocols. However, a significant positive correlation was found in PC9 between cervical mucus discharge, uterine tone, and pregnancy rate (*p* < 0.01), a relationship that was not observed in J-Synch6 (*p* > 0.05).

The effect of the number of hours in each THI category during the seven critical phases of the TAI program on estrus signs and pregnancy rate was analyzed by protocol (Table 4). In the J-Synch6 protocol, during Phases 1, 2, 3, and 4, each additional hour in Category 0 (THI < 72) increased the likelihood of estrus onset (*p* < 0.05), but had no effect on UT3, cervical mucus, or pregnancy. Additionally, in this protocol, each additional hour in Category 3 (THI ≥ 83) during Phase 3 had a negative effect on the probability of estrus onset (*p* < 0.05). In the PC9 protocol, no significant effects were observed. No significant association was found between the number of hours in the THI categories and positive pregnancy rate during Phases 5, 6, and 7.

## 4. Discussion

The efficacy of two estrus synchronization protocols in Gyr crossbred dairy cows was evaluated through estrus manifestation signs and pregnancy. No significant association was found between uterine tone (UT) and the synchronization protocols. However, there was a tendency toward a higher frequency of UT3 with similar values in both protocols (67.09% in PC9 and 66.25% in J-Synch6), suggesting a favorable inductive effect on uterine tonicity. A higher UT score (UT3) is associated with an optimal uterine condition for fertilization and embryo implantation, creating favorable conditions for early gestation maintenance. Navarro et al. [21] reported that the J-Synch protocol promotes greater uterine receptivity by extending the proestrus phase, potentially improving intrauterine conditions and reproductive success rates. However, PC9 in this study showed a similar uterine tone response, and the correlation with pregnancy rate was positive in both protocols. This highlights their effectiveness in synchronizing estrus in crossbred Gyr dairy cows.

The presence of cervical mucus during insemination is a reliable indicator of hormonal stimulation response, reproductive tract receptivity, and fertility in zebu cattle [22,23,24]. The absence of differences in cervical mucus frequency between protocols suggests similar effectiveness in stimulating mucus production, which is essential for sperm transport toward the oviduct, enhancing the likelihood of ovum fertilization [7,10].

Similarly, estrus detection by patch showed no significant differences between protocols. Although estrus detection is not required for FTAI programs, the manifestation of estrus behavior remains a valuable indicator of hormonal responsiveness and physiological readiness for insemination. The approximately 60% detection rate demonstrates both protocols’ ability to induce estrogenic activity and promote receptive behavior, as evidenced by significant patch decoloration from mounting activity. In zebu breeds like Gyr, estrus expression is particularly important due to its subtler and shorter duration compared to taurine breeds, complicating timely detection [7]. Despite similar estrus presence in both protocols, J-Synch6 resulted in a significantly larger follicular diameter compared to PC9 before insemination, likely due to its prolonged proestrus phase.

Previous studies on the J-Synch protocol have reported varying estrus expression rates. Macagno et al. [25] observed high estrus presence (85%), while Pilla-Campaña et al. [26] found 67% with J-Synch and 91% with an 8-day conventional protocol. Bó et al. [16] suggested that the prolonged proestrus phase in J-Synch offers advantages by optimizing dominant follicle development, improving oocyte quality, and promoting increased estrogen secretion, uterine tone, and estrus expression [15]. The variability in these results could be attributed to differences in environmental conditions, animal heterogeneity, and production management across studies [27]. In this study, J-Synch6 improved follicular diameter; however, this was not reflected in a significant increase in uterine tone, cervical mucus presence, or estrus expression. Adverse environmental conditions often pose significant challenges to normal reproductive activity in cattle [12]. In tropical environments, heat stress negatively impacts reproductive performance. Strategies that prolong the proestrus phase, such as the J-Synch protocol, may help mitigate these limitations by optimizing estrous cycle functionality [5].

The pregnancy rate was not significantly different between J-Synch6 (42.5%) and PC9 protocols (39.2%), suggesting a similar effectiveness in the estrus synchronization of crossbred Gyr dairy cows under tropical conditions. These pregnancy rates align with Reineri et al. [28], who reported 44.28% with J-Synch and 42.48% with a 7-day conventional protocol. However, they were lower than Pilla-Campaña et al. [26], who reported 56% with J-Synch and 54% with an 8-day conventional protocol. Sales et al. [14] emphasize that larger and well-developed preovulatory follicles are more likely to yield viable oocytes, improving pregnancy rates. The prolonged proestrus phase in J-Synch has been associated with increased estrogen secretion, promoting uterine receptivity and optimal conditions for fertilization and embryonic development [5]. However, in this study, no statistical difference was found compared to PC9.

The highest pregnancy rates were observed in cows with a corpus luteum (CL) at the start of the protocol. While CL presence was not statistically associated with estrus expression, it is a key indicator of normal ovarian cyclicity, which appears to enhance synchronization success and positively influence pregnancy outcomes. This aligns with Baruselli et al. [8], who reported that cyclic cows achieve better reproductive outcomes in FTAI programs. Conversely, cows without an initial CL may exhibit reduced cyclic activity or anestrus, limiting their hormonal response to synchronization treatments. These findings highlight the importance of rigorous selection of eligible cows for FTAI programs to improve reproductive rates, especially under tropical conditions. In both protocols, positive pregnancy outcomes were correlated with the presence of a CL, uterine tone, and estrus detection via patches. However, only in PC9 was cervical mucus presence significantly associated with pregnancy, suggesting that while mucus frequency was similar in both protocols, it may serve as a more reliable indicator of successful pregnancies in PC9 than in J-Synch6. Overall, the manifestation of estrus signs reflects the proper functional coordination of the hypothalamic–pituitary–ovarian axis, indicating optimal conditions for ovulation and implantation [29].

In tropical regions, the effectiveness of estrus synchronization protocols and FTAI programs is influenced by both environmental and animal-related factors [23]. High ambient temperatures and relative humidity significantly affect bovine homeostasis and comfort, as thermoregulatory efforts to counteract heat stress can impair both productive and reproductive efficiency [29,30]. The temperature–humidity index (THI) is a reliable tool for estimating heat stress risk in cattle by defining tolerance ranges and critical levels that may reduce reproductive capacity. In J-Synch6, during Phases 1, 2, 3, and 4, each additional hour in Category 0 (THI < 72) increased the likelihood of estrus onset. Higher estrus expression was associated with a greater number of hours spent in the absence of heat stress risk (Category 0 or THI < 72) during antral follicle emergence, the start of the previous estrous cycle, follicular growth, and pre-ovulatory follicle phases. Similarly, a higher estrus presence was linked to fewer hours under severe heat stress conditions (Category 3 or THI ≥ 83) on the day of AI (Phase 3, follicular growth phase). Many studies have demonstrated the detrimental effects of prolonged exposure to high temperatures on estrus expression and pregnancy rates in cattle [6]. Heat stress impacts the hormonal regulation of the estrous cycle and estrus manifestation signs [30,31,32]. It interferes with the hypothalamus’s ability to release GnRH, which in turn affects LH secretion, essential for ovulation and complete estrus expression [29,30]. In this study, the prolonged proestrus phase of the J-Synch protocol enhanced dominant follicle development, leading to a higher-quality oocyte with better conditions for successful fertilization and pregnancy [33]. Improved thermal comfort conditions potentially facilitated estrus expression due to the development of larger, more mature dominant follicles. In PC9, estrus expression was not affected by any THI category.

According to the literature, a THI above 72 indicates moderate heat stress in cattle, which can negatively affect reproductive efficiency [5]. Elevated THI levels have been shown to impair corpus luteum (CL) function, reducing progesterone production—a key hormone for maintaining early pregnancy [31]. However, in this study, the effect of THI during fertilization, implantation, and early fetal development phases appeared to be mitigated by the adaptability of crossbred Gyr cattle to tropical conditions. In tropical dairy cattle like Gyr, estrus signs are often less intense and are significantly influenced by environmental conditions, nutritional status, and herd management. A near-comfort thermal environment, with THI close to 72, seems to enhance reproductive responses. However, maintaining thermal stability and ensuring adequate CL functionality are crucial for optimizing estrus synchronization protocols [34]. In the conventional protocol, the administration of estradiol salts at CIDR removal induces positive feedback on GnRH, increasing the frequency and amplitude of LH pulses, which stimulates ovulation within a short timeframe. However, under heat stress conditions, follicular development may be incomplete, compromising estrus expression and fertilization success [35].

In this study, estrus signs and pregnancy rates were similar between the two protocols, despite comparable environmental conditions and heat stress risk. However, key differences emerged regarding the impact of THI on reproductive parameters: In J-Synch6, with a longer proestrus period, estrus expression was favored by the greater number of hours with THI < 72 (without stress), critical Phases 1, 2, 3, and 4. In PC9, UT3, cervical mucus discharge, estrus manifestation, and pregnancy rates were not significantly influenced by THI categories. However, a larger sample size might reveal more pronounced differences between protocols according to the level of influence of heat stress. Moreover, monitoring estrus expression and pregnancy rates over multiple periods with similar THI conditions could help clarify the sensitivity and strategic use of estrus synchronization protocols in the tropics. This analysis underscores the need for implementing heat stress management strategies in tropical livestock systems to optimize reproductive outcomes. Tailoring protocols to specific environmental conditions and ensuring thermal comfort could enhance the efficiency of FTAI programs under tropical climates.

## 5. Conclusions

The estrus signs and pregnancy rates in this study were similar between the two estrus synchronization protocols (J-Synch6 and PC9) in crossbred Gyr dairy cows, reinforcing their comparable effectiveness under tropical conditions. However, J-Synch6 demonstrated potential advantages in follicular development and estrus expression sensibility due to its prolonged proestrus period, showing a stronger relationship with lower THI values (no heat stress risk), a relationship not observed in PC9. In the context of climate change adaptation, weather monitoring could play a crucial role in planning FTAI programs, selecting appropriate synchronization protocols, and ultimately optimizing reproductive efficiency in tropical livestock systems.

## Figures and Tables

**Table 1:** Estrus manifestation signs, follicular diameter, and pregnancy rate of cows under two estrus synchronization protocols.

Protocol	* UT (%)			Cervical Mucus (%)	** Follicular Diameter (mm)	Estrus Rate (%)	Pregnancy Rate (%)
	UT1	UT2	UT3				
PC9	0 (0)	32.91 (26)	67.09 (53)	43.04 (34)	12.54 ± 3.43 ^b^	59.49 (47)	39.24 (31)
J-Synch6	1.25 (1)	32.50 (26)	66.25 (53)	45.00 (36)	14.47 ± 4.03 ^a^	60.00 (48)	42.50 (34)
*p*-value	0.79			0.80	0.02	0.95	0.68

PC9: Conventional protocol, J-Synch6: J-Synch protocol. Percentages and case counts are shown. Association analyzed using the Chi-square test (*p* < 0.05). * UT: uterine tone. ** Mean ± SD, comparison by ANOVA and *t*-test. Different superscript letters ^(a,b)^ in columns indicate significant differences at the *p* < 0.05 level.

**Table 2:** Estrus and pregnancy rates according to evaluation period, presence of cervical mucus, and presence of an initial corpus luteum.

Variable	Levels	Estrus (%)	Pregnancy (%)
Period	Period 1 (September–December)	47.6 (20)	38.1 (16)
	Period 2 (January–March)	53.6 (15)	42.9 (12)
	Period 3 (April–July)	68.9 (31)	44.4 (20)
	Period 4 (July–August)	65.9 (29)	38.6 (17)
	*p*-value	0.15	0.92
Cervical mucus	Present	68.1 (47)	49.3 (34)
	Absent	53.3 (48)	34.4 (31)
	*p*-value	0.06	0.06
Corpus luteum	Present	65.7 (44)	56.7 (38) ^a^
	Absent	55.4 (51)	29.3 (27) ^b^
	*p*-value	0.19	<0.01

Percentages and case counts are shown. Different superscript letters (a,b) in columns indicate a significant association based on the Chi-square test at the p < 0.01 level.

**Table 3:** Correlation between estrus signs, presence of an initial corpus luteum, and positive pregnancy in crossbred dairy Gyr cows subjected to two estrus synchronization protocols.

	PC9				J-Synch6			
	Cervical Mucus	Estrus Presence	Pregnancy	Corpus Luteum	Cervical Mucus	Estrus Presence	Pregnancy	Corpus Luteum
Uterine tone	0.391 (**)	0.355 (**)	0.397 (**)	0.102	0.180	0.447 (**)	0.297 (**)	0.147
	<0.001	<0.001	<0.001	0.373	0.110	<0.001	0.007	0.193
Cervical mucus		0.196	0.296 (**)	0.145		0.174	0.086	0.118
		0.083	0.008	0.202		0.122	0.446	0.295
Estrus presence			0.452 (**)	0.071			0.341 (**)	0.143
			<0.001	0.531			0.002	0.205
Pregnancy				0.265 (*)				0.268 (*)
				0.018				0.016

PC9: Conventional protocol, J-Synch6: J-Synch protocol. (**) Significant bilateral correlation at the *p* < 0.01 level. (*) Significant bilateral correlation at the *p* < 0.05 level.

**Table 4:** Coefficients of logistic regression between estrus signs and pregnancy rate in cows with THI across seven phases of the FTAI program, according to estrus synchronization protocols.

		PC9				J-Synch6			
Phases of FTAI Program	THI	UT3	Cervical Mucus	Estrus	Pregnancy	UT3	Cervical Mucus	Estrus	Pregnancy
Phase 1 (Antral follicle and emergence)	Cat. 0	0.005	−0.002	0.001	−0.001	0.005	−0.004	0.006 (*)	0.001
	Cat. 1	−0.006	0.003	0.003	0.001	−0.002	0.001	−0.006	0.001
	Cat. 2	−0.004	0.014	0.003	0.006	−0.012	0.011	−0.009	−0.002
	Cat. 3	−0.006	−0.002	−0.008	−0.003	−0.011	0.010	−0.010	−0.001
Phase 2 (Onset of the previous estrous cycle)	Cat. 0	0.007	−0.002	0.001	−0.001	0.007	−0.005	0.009 (*)	0.001
	Cat. 1	−0.007	0.002	0.005	0.002	−0.001	0.001	−0.006	0.001
	Cat. 2	−0.011	0.016	−0.002	0.005	−0.023	0.021	−0.020	−0.003
	Cat. 3	−0.008	−0.002	−0.011	−0.003	−0.015	0.013	−0.013	−0.001
Phase 3 (Follicular growth)	Cat. 0	0.047	−0.021	−0.004	−0.007	0.046	−0.033	0.056 (*)	−0.001
	Cat. 1	−0.082	0.231	0.204	0.119	−0.055	0.032	−0.097	0.008
	Cat. 2	−0.068	0.066	0.034	0.029	−0.068	0.050	−0.083	0.001
	Cat. 3	−0.072	−0.013	−0.037	−0.013	−0.075	0.058	−0.089 (*)	0.002
Phase 4 (Pre-ovulatory follicle)	Cat. 0	0.114	−0.037	−0.013	−0.013	0.094	−0.063	0.128 (*)	−0.006
	Cat. 1	−0.039	0.053	0.064	0.030	−0.006	−0.004	−0.034	0.008
	Cat. 2	0.052	−0.039	−0.170	−0.042	−0.123	0.133	−0.016	−0.038
	Cat. 3	−0.065	−0.082	−0.095	−0.050	−0.070	0.057	−0.076	0.001
Phase 5 (Ovulation and fertilization)	Cat. 0				0.005				0.001
	Cat. 1				−0.004				0.004
	Cat. 2				0.010				−0.014
	Cat. 3				−0.004				−0.022
Phase 6 (Recognition and implantation)	Cat. 0				0.001				0.001
	Cat. 1				0.002				0.001
	Cat. 2				−0.003				−0.002
	Cat. 3				−0.009				−0.003
Phase 7 (Early fetal development)	Cat. 0				0.001				0.001
	Cat. 1				0.001				−0.001
	Cat. 2				−0.004				−0.001
	Cat. 3				−0.005				−0.001

PC9: Conventional protocol, J-Synch6: J-Synch protocol. UT3: Uterine tone, THI: temperature–humidity index, Cat. 0: THI < 72 or no stress, Cat. 1: THI between 72 and 78 or alert state, Cat. 2: THI between 78 and 82 or danger state, and Cat. 3: THI ≥ 83 or emergency state. (*) Significance at the *p* < 0.05 level.

## Data Availability

The raw data supporting the conclusions of this article will be made available by the authors upon request.
